# Planning clinically relevant biomarker validation studies using the “number needed to treat” concept

**DOI:** 10.1186/s12967-016-0862-4

**Published:** 2016-05-04

**Authors:** Roger S. Day

**Affiliations:** Department of Biomedical Informatics, University of Pittsburgh, 5607 Baum Boulevard, Room 532, Pittsburgh, PA 15206 USA

**Keywords:** Clinical trial design, Biomarkers, Number needed to treat, Clinical utility, Bayes theorem

## Abstract

**Purpose:**

Despite an explosion of translational research to exploit biomarkers in diagnosis, prediction and prognosis, the impact of biomarkers on clinical practice has been limited. The elusiveness of clinical utility may partly originate when validation studies are planned, from a failure to articulate precisely how the biomarker, if successful, will improve clinical decision-making for patients. Clarifying what performance would suffice if the test is to improve medical care makes it possible to design meaningful validation studies. But methods for tackling this part of validation study design are undeveloped, because it demands uncomfortable judgments about the relative values of good and bad outcomes resulting from a medical decision.

**Methods:**

An unconventional use of “number needed to treat” (*NNT*) can structure communication for the trial design team, to elicit purely value-based outcome tradeoffs, conveyed as the endpoints of an *NNT* “discomfort range”. The study biostatistician can convert the endpoints into desired predictive values, providing criteria for designing a prospective validation study. Next, a novel “contra-Bayes” theorem converts those predictive values into target sensitivity and specificity criteria, to guide design of a retrospective validation study. Several examples demonstrate the approach.

**Conclusion:**

In practice, *NNT*-guided dialogues have contributed to validation study planning by tying it closely to specific patient-oriented translational goals. The ultimate payoff comes when the report of the completed study includes motivation in the form of a biomarker test framework directly reflecting the clinical decision challenge to be solved. Then readers will understand better what the biomarker test has to offer patients.

**Electronic supplementary material:**

The online version of this article (doi:10.1186/s12967-016-0862-4) contains supplementary material, which is available to authorized users.

## Background

### Motivation: disconnection between biomarker studies and clinical utility

Despite an explosion of research studies aiming to exploit biomarkers for eventual clinical application, their translation into impact on actual clinical practice has been minimal. Early detection biomarkers have mostly been unsuccessful, prognostic biomarkers are infrequently used in clinical decision making, surrogate endpoint biomarkers are rarely accepted for phase III clinical trials, and predictive biomarkers distinguishing patients who will benefit from a treatment have been difficult to validate. Tsilidis et al. [1] studied 98 meta-analyses of non-genetic biomarkers in association with cancer risk, finding that only 12 passed their criteria for believability. Hayes et al. [2] laments “the field of tumor biomarker research has been chaotic and haphazard, leading to many published papers in the peer-reviewed literature, but very few markers that truly have clinical utility”. Ransohoff asks [3] “Is it the normal stop-and-start of science? Or is there some systemic problem with the process that we currently use to discover and develop markers?” The skepticism has motivated individuals, committees and consortiums to identify problems, and promulgate guidelines for performing and reporting research, and for critical reviewing [4–14]. Ioannidis and Khoury [15], in lamenting a history of poor validation in “omics” biomarker studies, list six validation criteria. The last one listed is clinical utility: “Does the use of the discovered information improve clinical outcomes?” Indeed, the desirability of defining the intended clinical use (“actionability”) frequently receives mention, but guidance how to define it in practice is scarce. Consequently, most biomarker studies use statistical criteria, such as P values, hazard ratios, sensitivity and specificity, with only a murky relationship to the medical decisions and human health goals that physicians want to achieve with the biomarker. The planning, study design and presentation of biomarker validation study results usually omit concrete consideration of the desired improvements in decisions on behalf of patients.

Investigators should be able to state the intended clinical benefit: what specific improvement in decisions about treatment, screening or prevention the investigators hope the biomarker will achieve. The motivation of this work is to give clinical investigation teams a method to help articulate and clarify the goal for future patients. When such a goal cannot be stated, the justification of a study is rightfully called into question.

### Outline of approach and applications

The method introduced here produces quantitative criteria, chosen so that the ethical tradeoffs between false positives and false negatives are easy to visualize in concrete terms. A biomarker validation study justified by an articulated patient-relevant quantitative goal will have suitable design and sample sizes. After completing the study, a reader can compare that original justification for the design with the study results, making it clear whether the new test should be adopted. Utilizing this method should reduce pointless biomarker validation studies, improve relevance of worthy studies, ensure adequate power for the purpose, and sharpen the interpretation of results.

**Fig. 1:**
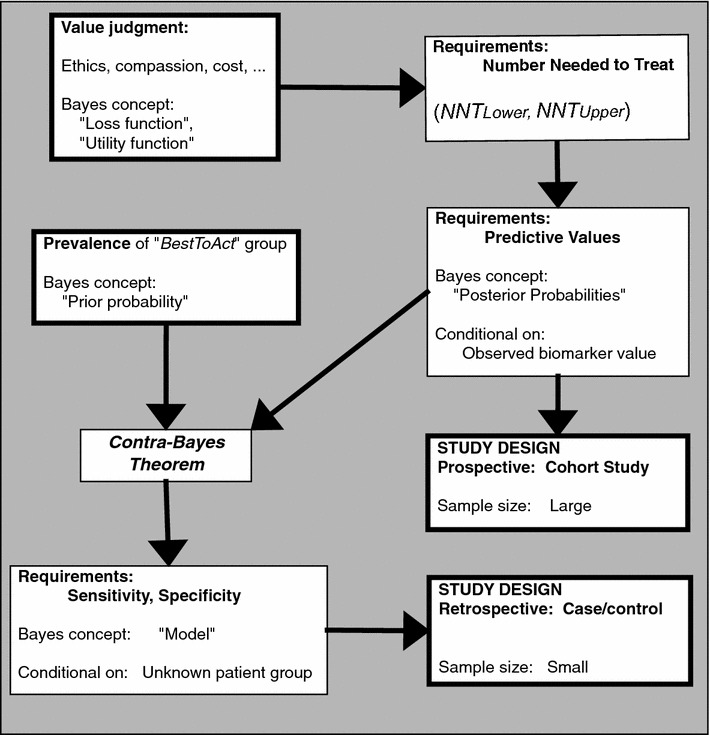
Roadmap for biomarker validation study design. Steps in the development of a study design for biomarker validation. *Upper left-hand boxes* judgments provided by clinical investigators. *Lower right-hand boxes* application of calculations of relevant quantities to the design of prospective (*middle right*) and retrospective (*lower right*) studies

The tools provided here can open communications within a trial design team to help them state performance criteria that correspond to genuine clinical usefulness. An unconventional use of the “number needed to treat” (*NNT*) concept provides a simple method to elicit clear specification of the intended medical use of the biomarker. An *NNT* “discomfort range” helps define the needed predictive values, providing meaningful criteria for the design of a prospective validation study. To guide the design of a retrospective validation study, a “contra-Bayes” theorem converts the predictive values into minimum requirements for sensitivity and specificity. Figure 1 and Table 1 present overviews. Several examples from cancer biomarker research illustrate the benefits and some remaining challenges. Interactive web pages driven by open-source software deliver easily accessible guidance.

**Table 1:** Steps in planning a retrospective biomarker validation study

	Stepping stone	Question format
0	Classification rule development	(Outside the scope of this article)
1	Defining the clinical scenario	Who are the patients, what are the clinical options?
2	Principal goal	What *NNT* s for the *BestToAct* and *BestToWait* groups would make the decision clear-cut?
3	Clinical benefit	Specifically how will patients be helped by a test that achieves these *NNT’s*?
4	Classification performance needed	What predictive values do these *NNT’s* correspond to?
5	Prospective study requirements	Given these *NNT’s*, how large should a prospective study be, and how long the follow-up?
6	Retrospective study requirements	Given a prevalence, what sensitivity and specificity do we hope for, and what should the sample sizes be to estimate them sufficiently?

“Scaffolding for NNT-guided protocol design” illustrates these study planning steps for a specific study

**Table 2:** Connecting sensitivity, specificity, and prevalence, to predictive values and *NNT* values

							
Sensitivity	0.50	0.60	0.70	0.80	0.90	1.00	
Specificity	0.50	0.60	0.70	0.80	0.90	1.00	
*PPV*	0.50	0.60	0.70	0.80	0.90	1.00	Prevalence = 0.5 (see Fig. 3a)
*NPV*	0.50	0.60	0.70	0.80	0.90	1.00	Prevalence = 0.5 (see Fig. 3a)
*NNT*_*Pos*_	2.0	1.7	1.4	1.3	1.1	1.00	Prevalence = 0.5 (see Fig. 3a)
*NNT*_*Neg*_	2.0	2.5	3.3	5.0	10.0	Inf	Prevalence = 0.5 (see Fig. 3a)
*PPV*	0.05	0.07	0.11	0.17	0.32	1.00	Prevalence = 0.05 (see Fig. 3b, c)
*NPV*	0.95	0.97	0.98	0.99	0.99	1.00	Prevalence = 0.05 (see Fig. 3b, c)
*NNT*_*Pos*_	20.0	13.7	9.1	5.8	3.1	1.00	Prevalence = 0.05 (see Fig. 3b, c)
*NNT*_*Neg*_	20.0	29.5	45.3	77.0	172.0	Inf	Prevalence = 0.05 (see Fig. 3b, c)

Circled letters refer to points labeled in Fig. 3. Example: for column *D*, if the test sensitivity and specificity both equal 0.80, and the prevalence is 0.05, then the predictive values for the test are respectively 0.17 and 0.99 (point *D* graphed in Fig. 3b), and the *NNT* values in the positive and negative test groups are respectively 5.8 and 77.0 (point *D* graphed in Fig. 3c)

## Methods

### Setting and terminology for medical decision-making and testing

Medicine requires making decisions in the face of imperfect information. The Bayesian framework [16] is especially well suited for medical decision-making, with a long history [17–19]. We consider the simplest binary medical decision: an action to take or refrain from. The action contemplated may be a medical treatment, a costly or risky diagnostic procedure, an onerous, costly monitoring schedule for early detection, or enrolling a patient onto a clinical trial. The following terminology covers these cases.

Suppose some biological characteristic of a patient would determine our choice between acting or waiting if we knew its status: either *BestToAct* or *BestToWait*. Initial knowledge or belief about the patient’s status is represented by a “prior probability” Pr(*BestToAct*), which could express a precise estimate or a subjective opinion. When there is treatment controversy, Pr(*BestToAct*) is too far from certainty (one or zero) to make the best clinical decision clear to most physicians.

The intention is that some biomarker test yielding a positive (*Pos*) or negative (*Neg*) result will reveal something about the patient status, so that knowing the test result updates our knowledge, expressed by moving Pr(*BestToAct*) up or down using Bayes Theorem. Now, one decision challenge is replaced by two: for *Pos* and for *Neg* patients. The hope is that they will have clear and opposite decisions. (What “patient status” refers to receives some discussion later).

#### *NNT* and performance criteria for biomarker validation clinical trials

Laupacis et al. [20] introduced “number needed to treat”, *NNT,* to summarize results of antihypertensive therapy to reduce hypertension-related adverse events: how many patients needed to be treated in order to benefit one patient. They reported *NNT* = *NNT*_*Neg*_ = 17 in patients without target-organ damage, and *NNT* = *NNT*_*Pos*_ = 7 with damage. The subscripts “*Pos*” and “*Neg*” come about by thinking of target-organ damage as “biomarker” test, positive if present, negative if absent. In that setting, the two *NNT* values described the size of the treatment effect in clinically relevant terms, for the “*Pos*” and “*Neg*” patient groups.

To use a *NNT* value in deciding whether to treat a patient, a physician needs to combine it with value judgments weighing harms versus benefits accruing to different people. If *NNT* patients are all treated, and one benefits, there are *NNT* − 1 “victims” not helped by the treatment. If none are treated, the *NNT* − 1 who should not receive the treatment are saved from it, but the one patient who would have been helped will not receive the help. Which of these two collective results on the *NNT* patients is best may be fairly termed an ethical judgment. (For convenience, this paper uses the term “ethical” in reference to value judgment tradeoffs, without implying that any particular action would deserve the epithet “unethical”).

**Fig. 2:**
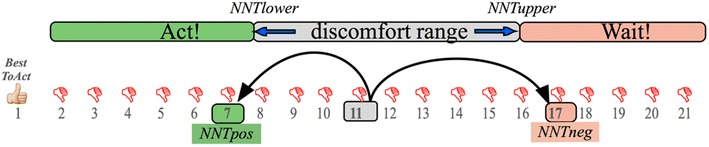
*NNT* and clinical decisions. On the *left* is a single patient for whom it is “*Best to act*”, indicated by the “thumb’s up” *sign*. The *horizontal* scale refers to the number of patients needed to treat (*NNT*) in order to help one. The range [*NNT*_*Lower*_, *NNT*_*Upper*_] should describe a range of discomfort with either decision, *Act* or *Wait*

Consider the example in Fig. 2. For *NNT* between 8 and 16, some medical decision is ethically uncomfortable; treating all patients (“*Act*”) entails treating too many who do not need it, but withholding treatment for all (“*Wait*”) misses too many opportunities to help some of the patients. We call the interval (*NNT*_*Lower*_, *NNT*_*Upper*_) = (8, 16) the *NNT* discomfort range. Suppose the observed *NNT* for all the patients is 11. Treating all 11 means helping one (a *BestToAct* patient), but subjecting the other ten to treatment without benefit (*BestToWait*). Being within the hypothetical discomfort range, this number implies a clinical decision dilemma. Suppose we now have a test separating patients into a *Positive* group, with *NNT* = *NNT*_*Pos*_ = 7 and a *Negative* group with *NNT* = *NNT*_*Neg*_ = 17. Both values are outside the discomfort range. Then the physician will comfortably treat a patient in the *Pos* group, since eight is greater than *NNT*_*Pos*_ = 7. Since the physician also judges that treating more than 16 (*NNT*_*Upper*_) to help one *BestToAct* patient is too much unnecessary overtreatment, then they will comfortably refrain from treating a patient in the *Neg* group. The test information usefully informs the treatment decision. Knowing the test information, combining the objective knowledge *NNT*_*Pos*_ and *NNT*_*Neg*_ with the subjective discomfort boundaries *NNT*_*Lower*_ and *NNT*_*Upper*_ converts an uncomfortable clinical decision into a clear decision for both *Pos* and *Neg* subgroups.

This paper proposes eliciting the *NNT* discomfort range, choosing desired values outside this range for *NNT*_*Pos*_ and *NNT*_*Neg*_ in the test-positive and test-negative subgroups, and leveraging those values to guide the study design. This exercise helps the study team determine a relevant patient population and rules for selecting specimens, and ensure that the study’s eventual results will have utilitarian interpretability for guiding clinical decisions.

To elicit the *NNT* discomfort range, we strip physician preferences down to the essentials. One guides the *NNT* respondent, typically the clinical principal investigator, to imagine a clinic schedule of patients, together with the certain knowledge that, if treated, exactly one will receive the benefit hoped for. With this scenario, all uncertainties are removed, and all outcomes are known; the only thing unknown is which of the *NNT* patients will be the sole beneficiary. Framing the problem in terms of a fixed number of patients with fixed outcomes rather than probabilities or proportions circumvents the well-known documented numeracy deficiencies that plague even medical researchers [21, 22].

The immediate goal is to elicit a pure judgment about the value tradeoffs. With fixed, non-probabilistic outcomes, making these judgments becomes simpler. The final goal is to describe desired performance requirements for a genuinely helpful clinical test. These requirements will feed the design of a meaningful clinical study.

Declaring values for *NNT*_*Lower*_ and *NNT*_*Upper*_ requires courage, because it entails exposing one’s subjective valuations trading off the benefits of helping versus the costs of overtreating. However, compared to declaring either probabilities or abstract disembodied “utility” tradeoffs, the concrete visualization of a group of patients and their outcomes is more likely to produce a meaningful answer. The burden of imagination is not so great, because there are no probabilities to guess at or interpret. The totality of the outcomes is fixed; not just the benefits but also any costs or side effects associated with treating the patients would already be taken into account in the imagination of the *NNT* respondent, just as they are when making a clinical decision for a real patient.

The *NNT* framework helps to ground in the real world any discussion of what would make a clinical test truly useful. Prior to discussing an *NNT* discomfort zone, one respondent stated that *PPV* = 15 % and *NPV* = 70 % would be sufficient for a useful test, perhaps deliberately setting a low bar. Many people might notice that these values are unambitious. It may not be instantly obvious that they do not even make sense. That becomes clear after translating to *NNT*_*Lower*_ = 1/0.15 = 6.7 and *NNT*_*Upper*_ = 1/(1 − 0.70) = 3.3, since *NNT*_*Lower*_ must be smaller than *NNT*_*Upper*_. In another setting, the stated desired performance was sensitivity = 30 %, specificity = 70 %, a performance achievable with a weighted random coin flip. Thus, elicitations of desired test performance not using *NNT* ideas can be problematic even among researchers. Brawley’s discussions [23] of the ethical issues in radical prostatectomy and screening in prostate cancer are instructional. He uses observed *NNT* values explicitly to shed light on whether the *NNT* is too large to warrant treatment or screening; in our terms, bigger than *NNT*_*Upper*_. Anyone capable of judging whether an observed *NNT* is too large or too small for a comfortable decision should also be capable of setting an *NNT* discomfort range.

The study contemplated is to validate a biomarker test separating patients into two subgroups, *Pos* and *Neg.* The desired clinical performance is described by

1 $$NNT_{Pos} < \underbrace {{NNT_{Lower} < NNT_{Upper} }}_{\text{discomfort range}} < NNT_{Neg} .$$

If the study finds that the biomarker test achieves the outer inequalities, then the test has a good opportunity to improve patient care; otherwise, little chance. Without the biomarker, the actual *NNT* for the entire group of subjects might be lower than *NNT*_*Lower*_, higher than *NNT*_*Upper*_, or in between. Each of these three scenarios presents a distinct opportunity to improve clinical practice. For the current exposition, we focus on the “in between” case, illustrated in Fig. 2.

### Mapping from NNT range to predictive values for prospective study design

*NNT* maps directly to the familiar concepts positive and negative predictive values (*PPV* and *NPV*). We visualize the aftermath of successful validation studies, when a useful test has been developed, with *NNT*_*Pos*_ and *NNT*_*Neg*_ as the estimated *NNT* values for *Pos* and *Neg* patients. Then in a group of *NNT*_*Pos*_ treated test-positive patients, one of them will benefit. This patient is a “true positive”, so the positive predictive value, *PPV,* is 1/*NNT*_*Pos*_. Among *NNT*_*Neg*_ patients with *Neg* results, none of whom are treated, one of them would have benefitted if treated, constituting a “false negative”. Therefore *NPV* is 1 − 1/*NNT*_*Neg*_. Combining the requirements for a useful test (Eq. 1) with these mappings, we need

2 $$PPV > 1/NNT_{Lower} ,\quad NPV > 1{-}1/NNT_{Upper} .$$

To plan a prospective validation study, one chooses the desired precision for estimating *PPV* and *NPV* and confirming *NNT*_*Pos*_ < *NNT*_*Lower*_ and *NNT*_*Upper*_ < *NNT*_*Neg*_ to a desired level of confidence. Then the usual biostatistical considerations determine the minimum sizes of the *Pos* and *Neg* subgroups, for whatever standard or nonstandard study design is desired. Because the study is prospective, only the overall sample size is controlled; the proportions of *Pos* and *Neg* patients are not (unless the test is quantitative and the categories are adjustable by moving a continuous cutoff, not discussed in this paper).

### Retrospective studies: mapping from desired predictive values to desired sensitivity and specificity

Because of the cost, size and extended duration of a prospective study, a retrospective case–control study usually comes first. Assembling a sample of cases for a retrospective case–control study means identifying people who, in hindsight, are known to be in the *BestToAct* category: patients who were not treated but (we now know) should have been treated. The controls are from the *BestToWait* category: untreated patients with good outcomes, or else treated patients who were not helped.

A key design challenge is to decide the sample sizes for selected cases and controls, the *BestToAct* and *BestToWait* patients. The case–control study will then determine the test status, *Pos* or *Neg,* for each subject. These data will provide the estimates of the sensitivity (*SN)* and specificity (*SP*). To choose sample sizes for the validation study, what *SN* and *SP* values correspond to the clinical usefulness we seek?

Bayes theorem uses *SN* and *SP*, together with the prevalence, to generate *PPV* and *NPV.* However, we now need to go in reverse, from required *PPV* and *NPV* to required *SN* and *SP*. In terms of the prior odds, *Odds* = Pr(*BestToAct*)/Pr(*BestToWait*), the “positive predictive odds” *PPO* = *PPV/*(*1* − *PPV*), and the “negative predictive odds” *NPO* = *NPV/*(*1* − *NPV*), we have a “contra-Bayes” theorem:

3 $$\begin{aligned} SP = specificity = \frac{PPO - Odds}{{PPO - NPO^{{{-}1}} }} \hfill \\ SN = sensitivity = \frac{{NPO - Odds^{{{-}1}} }}{{NPO - PPO^{{{-}1}} }} \hfill \\ \end{aligned}$$

(This has a meaningful solution whenever the positive and negative test results change the prior odds in the expected directions: *PPO* > *Odds* > *NPO*^−1^. Additional file 1 demonstrates verification of Eq. 3 by simple algebraic substitution and simplification.)

**Fig. 3:**
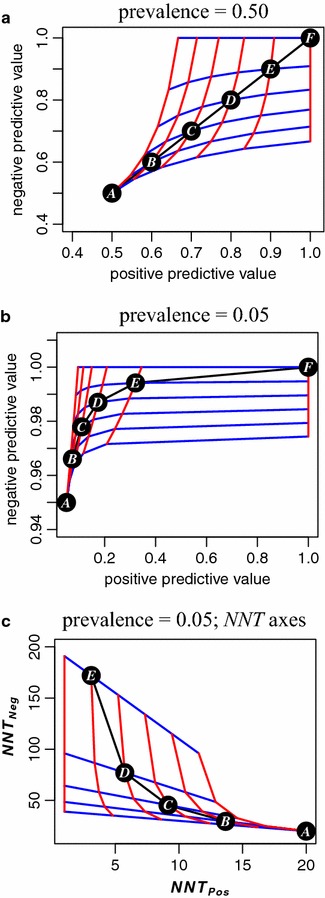
Contra-Bayes mapping from predictive values to sensitivity and specificity. See text and Table 2 for details. The points labeled *A* through *F* correspond to sensitivities and specificities given in Table 2 lines 1 and 2. **a** Values from Table 2 lines 3 through 6; **b**, **c** values from Table 2 lines 7 through 10. **c** uses NNT axes in place of predictive value axes. An interactive web application “shinyContraBayes” [29] is available, and also embedded in a more comprehensive application “shinyCombinePlots” [30]

Figure 3 shows regions where the solution to Eq. 3 yields a feasible sensitivity/specificity pair. The left-side boundary of the feasible region is defined by sensitivity = prevalence; the bottom boundary is defined by specificity = 1 − prevalence. The steeply slanted red lines are contours of constant sensitivity. The gently slanted blue lines are contours of constant specificity.

The black line connecting the lettered labels marks points where the sensitivity equals the specificity. See Table 2 for values at these points. Point *A* corresponds to a purely random “test”. Point *F* corresponds to a perfect test. The left-side boundary of the feasible region is defined by sensitivity = prevalence; the bottom boundary is defined by specificity = 1 − prevalence. For example, in Fig. 3c, beginning at point *D* (sensitivity = specificity = 0.80), climbing the steep red line increases the specificity only, and improves *NNT*_*Neg*_ over 150, while improving *NNT*_*Pos*_ negligibly.

## Results

### Scaffolding for NNT-guided protocol design

Table 1 presents key questions and steps along the path to a biomarker validation study design. A consultation in which this author participated illustrates these steps, in a relatively simple setting. *Step 1* Clinical scenario: Prognosis of cutaneous T cell lymphoma (CTCL). In early stages of CTCL, patients (Stages IA-IIA) usually do well and have slowly progressive disease, which does not require aggressive therapy associated with substantial side effects. However, about 15 % of these patients have unexpected progressive course and rapid demise. *Step 2* Principal goal: To identify, among patients who are diagnosed with early stage CTCL, who should receive the aggressive therapy immediately. *Step 3* Clinical benefit: A biomarker progression risk model that is able to classify patients into high and low risk groups will enable personalized and more aggressive therapy for the patients at highest risk for progression. *Step 4* Classification performance needed: Regarding more aggressive therapy upfront, the PI stipulates that the classifier will have clinical utility if the “number needed to treat” (*NNT*) is less than *NNT*_*Lower*_ = 2 in the test-positive patients, and greater than *NNT*_*Upper*_ = 30 in the test-negative patients. Then two patients testing positive will need to receive aggressive treatment upfront in order to treat one patient who otherwise would suffer later rapid progressive CTCL, while in the test-negative patients one would have to treat an unacceptably high 30 patients to treat one patient in advance of progressive CTCL. This performance should suffice to create a clinical consensus supporting using the test for clinical decisions. *Step 5* Prospective study requirements: The values *NNT*_*Lower*_ = 2 and *NNT*_*Upper*_ = 30 correspond to the positive predictive value *PPV* = 50 % = 1/*NNT*_*Lower*_, and the negative predictive value *NPV* = 97 % = 1 − 1/*NNT*_*Upper*_. We will be able to recruit 40 patients in this early-stage group, over 3 years, with a minimum of 2 years follow-up thereafter. If the test divides the 40 patients into roughly 25 % positive and 75 % negative, and the estimates match the hoped-for values 2 and 30, the confidence intervals would be 19–81 % for *PPV*, and 83–100 % for *NPV*, or equivalently [24] 1.23–5.35 for *NNT*_*Pos*_, and 5.81–1180 for *NNT*_*Neg*_. The very wide confidence interval for *PPV* is due to the low sample size and low prevalence combined with the low value for *NNT*_*Pos*_, which is strongly weighted towards avoiding unnecessary aggressive therapy. To obtain a more accurate and independent estimate of *PPV*, we also plan a retrospective study. *Step 6* Retrospective study requirements: Combining *PPV* and *NPV* with an incidence of rapid progression of 15 %, the required sensitivity (*SN*) and specificity (*SP*) are 83.3 and 85.3 %, respectively (contra-Bayes Theorem). To get a sense of the accuracy of anticipated estimates in the retrospective (case/control) portion of the study, we consider anticipated results for samples sizes 22 cases (the entire complement of early stage CTCL who rapidly progressed) and 40 controls. For example, if the estimates *SN* = 18/22 = 82 % and *SP* = 34/40 = 85 % are observed, then the corresponding confidence intervals will be 60–95 % for *SN*, and 70–94 % for *SP*, and Bayes predictive intervals will be (1.4, 2.7) for *NNT*_*Pos*_, and (16.4, 87.8) for *NNT*_*Neg*_. (These intervals derive from assuming independent Jeffreys priors for *SN* and *SP*, sampling from joint independent posteriors incorporating the anticipated results, and applying Bayes theorem).

Steps 1 through 3 of the *NNT* scaffold force us to state the clinical dilemma that the biomarker is intended to help resolve. Step 4 is the critical ethics-balancing step that the *NNT*-based visualization supports. A reader might find fault with the clinical judgments expressed in steps 1 through 4, and that is exactly the point: exhibiting answers to these questions explicitly facilitates meaningful discussion and critique. Steps 5 and 6 provide the statistical designs. Taken together, the scaffold allows us to judge whether these prospective and retrospective study designs adequately address the dilemma. Because of the *NNT* focus, after the study finishes, a published report can describe the potential for impact on clinical care in clinically visualizable and meaningful terms.

Several ways to improve the above statistical design and analysis plan come to mind, for example doing a sensitivity analysis with regard to assumptions, polling multiple clinicians for their personal *NNT*_*Lower*_ and *NNT*_*Upper*_ values, incorporating risk factors, and supplementing with power calculations. These issues are beyond the scope of this article.

### Thinking critically about what constitutes a BestToAct patient

An application of the *NNT* method to an endometrial cancer biomarker study illustrates how *NNT* thinking clarifies whether a new biomarker test has a realistic goal for helping patients. Defining what constitutes a *BestToAct* patient is the challenge that brings this question to focus. It affects whether a biomarker study should be done at all.

A biomarker study to predict regional metastasis of endometrial cancer was under discussion. The overall purpose was to guide community oncologists and surgeons concerning whether to refer a case to a gynecologic oncology surgeon, a specialist with in-depth experience with surgical staging of endometrial cancer. An observational study had suggested that referral for intensive surgical staging could improve survival in high-risk patients [25]. However, around 75 % of endometrial cancer patients are Stage 1, generally curable with surgery alone; less than 20 % overall die of the disease before 5 years; and the clinical value of other treatment options for preventing or treating recurrent disease has little high-quality evidence [26]. Therefore room for improving patient outcomes is somewhat limited.

Most women diagnosed with endometrial cancer receive their diagnosis through oncologists who are not specialists in gynecological oncology. Most of these are cured by primary surgery performed by general surgeons or general cancer surgeons. However, some of these patients have nodal metastasis outside the region that the nonspecialist surgeon would be likely to examine, but within a region that extensive surgical staging might discover.

In a small proportion of cases, community oncologists make referral to gynecologic cancer surgery specialists. Perhaps a biomarker test would guide them to referral more often. The *NNT* perspective can help determine whether a biomarker study to identify patients at high risk for metastasis should proceed, because it impels the clinical researcher to examine how such a test might benefit patients. One cannot consider the value of a “true positive” in isolation from subsequent decisions and outcomes. If a test is positive, and leads to an “action” of referral for extensive surgical staging, what happens next? Patients may belong to one of these categories:

1. *No disease discoverable by extensive staging*.
2. *Disease discoverable during excision of the primary in a community setting*.
3. *Disease discoverable by extensive staging only*.

To apply the *NNT* view, we again imagine a collection of *NNT* patients who will all be referred, or none referred, for surgical staging, with one patient in category #3, our tentative definition of *BestToAct*. An experienced gynecologic oncologist can imagine the range and frequency of negative consequences from the referral and extensive surgery to patients in categories 1 and 2. A harder task is to project the benefit to the one patient whose specialized surgery reveals metastasis. This is because the third group further divides into:

#3a *Patients whose subsequent treatment will not change despite the finding.*
#3b *Patients whose subsequent treatment will intensify, but without patient benefit*.
#3c *Patients whose subsequent treatment will intensify, and the patient will benefit*.

Therefore the choice whether to refer all *NNT* patients is not a pure ethical choice; to justify the decision, we still have to consider the probability that the category #3 patient is in category #3c. If the chance that the one group 3 patient is of type 3c is small, then one would demand a small *NNT.* The goal of eliciting *NNT* level representing a pure ethical assessment is not achieved yet; a probability is still involved.

Only a patient in group #3c will benefit, while the detriment of subjecting the other patients to more extensive surgery seems the same in groups #1, 2, 3a, or 3b. A reasonable change, therefore, is to reframe the question so that, of the *NNT* patients, exactly one is in category #3c, our revised definition of *BestToAct*. Then a larger *NNT* is acceptable, because the benefit to that patient is definite. However, the “prevalence”, the probability of being in the group that actually benefits, is much smaller. Recall that for designing a retrospective study, the contra-Bayes theorem requires this prevalence. It may be much easier to estimate the prevalence of an intermediate outcome like #3 than a truly relevant outcome like #3c. In this example, arguably an inaccurate estimate of the clinically relevant prevalence is better than an accurate estimate of a prevalence irrelevant to patient benefit. The goal, after all, is to deliver real benefits to patients, not just to generate usage of tertiary surgeries. To target a biomarker test at the achievement of real clinical benefit, the one patient who is the beneficiary among the *NNT* patients must truly benefit. If this principle guides the steps of the *NNT* scaffold and resulting discussion of *NNT*_*Lower*_ and *NNT*_*Upper*_, sometimes a compelling conclusion will be that the biomarker validation study contemplated has no chance of delivering a clinical improvement.

## Discussion and conclusion

Open-source software to support the process described here is freely available as a published R package NNTbiomarker [27], and also for immediate interactive use: shinyElicit [28], shinyContraBayes [29], shinyCombinePlots [30], and shinyAE [31].

Upon reporting completed trial results, the *NNT* method suggests comparing estimates of *NNT*_*Pos*_ and *NNT*_*Neg*_ to the edges of the discomfort zone, which should have been declared when the study was planned. Therefore, confidence intervals or predictive intervals for anticipated *NNT*_*Pos*_ and *NNT*_*Neg*_ results are clinically relevant. P values for testing a “no-difference” null hypothesis or a bioequivalence null hypothesis have their place, but are remote from communicating the biomarker’s clinical utility potential.

The application of *NNT* elicitation to develop objectives for biomarker studies sometimes involves challenging complications. A subset of them touch on timing of sample acquisition for biomarker assessment. Consider selecting samples when the setting is such that “*BestToAct*” samples represent occurrences of a future event that one wants to prevent. Frequently, the availability of convenience samples taken at the time of the future event impels investigators to use those samples as “cases”. This is problematic, because that time point does not coincide with the time point at which the biomarker would be used for the guiding medical decision, typically years earlier. If we are lucky, the biomarker does not change over time (for example, it may define a separate etiology and so be present from inception of the disease). Then a study confirming a biomarker using convenience samples from around the time of the event would validly reflect its usefulness for clinical practice, even though the time the *Act/Wait* clinical decision must be made is much earlier. However, the biomarker may appear gradually as the disease develops, prior to diagnosis but after the intended time of clinical decision. Then even a very strong positive predictive value for a measurement just prior to the event we want to prevent or prepare for may be useless; the biomarker would not yet be positive when the decision had to be made. This distinction, though obvious, is frequently overlooked. Even if the biomarker is present from the beginning, the sample defining the the patient group (“it would have been *BestToAct/BestToWait”*) is sometimes the same sample used to assess the biomarker. Patient’s classification and the patient’s biomarker value will have an extra spurious source of correlation due to variation from spatial sampling of tissue. Banked samples can eliminate this risk. For example, the Oncotype DX^®^ test was developed on banked samples, using microarray technology that was sufficiently accurate on paraffin-embedded primary cancer tissue. This tissue hailed from primary surgery, a time point in the clinical history near where decisions about adjuvant treatment would be made.

A related dilemma occurs when the desire is to develop a predictor for early-stage patients, but the available samples are from advanced-stage patients. One might also deliberately select advanced-stage patients, hoping that the signal we seek will be that much stronger in advanced-stage patients, so a good biomarker might be detected with a smaller sample size. This may be wishful thinking. Any step causing the study’s setting to differ from the hoped-for clinical decision setting where we want to deploy to test will risk irrelevancy. The *NNT* perspective puts the focus on the patients one hopes eventually to help with the test. This focus can prevent questionable research strategies.

This article only deals with binary biomarkers. We have also studied the *NNT* method in the context of continuous-valued biomarkers, specifically gene expression tests in breast cancer. The topic is an important extension, but beyond the scope of this article. A visual tool to examine consequences of treating for each recurrence score value is available [31]; the display is elaborated with information on adverse event outcomes as well.

The *NNT*-based approach presented here bears resemblance to the traditional threshold-based clinical decision methods [17, 19]. However, the approach here differs in two ways: by using *NNT* to circumvent subjective probability-based inputs, and by using two thresholds *NNT*_*Lower*_ and *NNT*_*Upper*_, rather than one. Two thresholds are necessary for defining the required operating characteristics of the study designs, as we have seen.

Sinclair et al. [19] studied *NNT* in the context of treating to prevent a bad outcome (“target event”, TE). They define a threshold for *NNT*, below which the preventive treatment should be given despite adverse events (AE) from the treatment. They show how to calculate the threshold from various costs and relative values. The proposal here shares some of this spirit, with important differences. First, our purpose here is to guide design of a clinical trial, not to evaluate an established treatment. Second, our use of *NNT* is to craft a story to elicit the relative value of the outcomes, which in the previous framework is just assumed known (TE relative to AE). Third, the choice of terminology in our framework allows it to apply across a wide variety of types of clinical decisions, not just preventing TEs.

Individual patients have their own relative valuations of the outcomes, which may differ in the test deployment situation from the idealization of the investigator planning the test validation study years earlier. To personalize each medical decision, honoring each patient’s individual circumstance and personality, is part of the art of medicine. As important as these considerations are, they cannot help in the study design phase; at that stage, thinking about patients in the abstract is necessary. It is certainly superior to thinking about statistical criteria remote from patient benefits and risks.

The impact of biomarkers on clinical medicine has been disappointing despite the extraordinary advance of molecular medicine and sophisticated high-throughput assays. The technique explored in this paper can contribute to the translational research process, guiding us towards interventions of real clinical benefit, by bringing concrete assessment of ethical tradeoffs to the definition of clinical utility.

## Additional file

10.1186/s12967-016-0862-4 Verification of the contra-Bayes theorem.

## Abbreviations

*NNT*: number needed to treat
TE: target event
AE: adverse event
ROC: receiver operation characteristics
RS: recurrence score
SN: sensitivity
SP: specificity
PPO: positive predictive odds
NPO: negative predictive odds
