# Supplementary material for: Planning clinically relevant biomarker validation studies using the “number needed to treat” concept
Source: J Transl Med. 2016 May 4;14:117. doi: 10.1186/s12967-016-0862-4 (PMC4857295; doi:10.1186/s12967-016-0862-4)
Supplement: Supplementary file 1 — 10.1186/s12967-016-0862-4 Verification of the contra-Bayes theorem. [file 12967_2016_862_MOESM1_ESM.docx]

# Supplementary Document 1

# Verification of the contra-Bayes theorem

From Bayes theorem.

$PPO=\frac{PPV}{1-PPV}=\frac{SN\times prev}{(1-SP)\times(1-prev)}=\frac{SN}{1-SP}\times Odds$.

Equation 3 provides the expressions for SN and SP:

$$\begin{matrix} SP=specificity=\frac{PPO-Odds}{PPO-NPO^{-1}}, \\ SN=sensitivity=\frac{NPO-Odds^{-1}}{NPO-PPO^{-1}}. \end{matrix}$$

Inserting these values into

$$\begin{matrix} \frac{SN}{1-SP}\times Odds=\frac{\left(NPO-Odds^{-1}\right)\left(NPO-PPO^{-1}\right)^{-1}}{\left(Odds-NPO^{-1}\right)\left(PPO-NPO^{-1}\right)^{-1}}\times Odds \\ =\frac{\left(NPO-Odds^{-1}\right)\left(PPO-NPO^{-1}\right)}{\left(Odds-NPO^{-1}\right)\left(NPO-PPO^{-1}\right)}\times Odds \\ =\frac{NPO\times PPO\times Odds-Odds-PPO+NPO^{-1}}{NPO\times Odds-PPO^{-1}\times Odds-1+NPO^{-1}PPO^{-1}} \\ =\frac{PPO\times(NPO\times Odds-Odds\times PPO^{-1}-1+NPO^{-1}PPO^{-1})}{NPO\times Odds-PPO^{-1}\times Odds-1+NPO^{-1}PPO^{-1}} \\ =PPO \end{matrix}$$

and likewise for *NPO*.
